# Pre-Op Hydronephrosis Predicts Outcomes in Patients Receiving Robot-Assisted Radical Cystectomy

**DOI:** 10.3390/cancers16162826

**Published:** 2024-08-12

**Authors:** Chris Ho-Ming Wong, Ivan Ching-Ho Ko, David Ka-Wai Leung, Seok Ho Kang, Kousuke Kitamura, Shigeo Horie, Satoru Muto, Chikara Ohyama, Shingo Hatakeyama, Manish Patel, Cheung-Kuang Yang, Kittinut Kijvikai, Ji Youl Lee, Hai-Ge Chen, Rui-Yun Zhang, Tian-Xin Lin, Lui Shiong Lee, Jeremy Yuen-Chun Teoh, Eddie Chan

**Affiliations:** 1 S.H. Ho Urology Centre, Department of Surgery, Faculty of Medicine, The Chinese University of Hong Kong, Hong Kong, China; 2 Department of Urology, Korea University Anam Hospital, Seoul 02841, Republic of Korea; 3 Department of Urology, Graduate School of Medicine, Juntendo University, Tokyo 113-8421, Japans-muto@juntendo.ac.jp (S.M.); 4 Department of Urology, Hirosaki University, Hirosaki 036-8561, Japan; 5 Department of Urology, The University of Sydney, Camperdown, NSW 2006, Australia; mpatel@med.usyd.edu.au; 6 Department of Urology, Taichung Veterans General Hospital, Taichung 40705, Taiwan, China; 7 Department of Urology, Ramathibodi Hospital, Mahidol University, Nakhon Pathom 10400, Thailand; 8 Department of Urology, Catholic University of Korea, Seoul 07345, Republic of Korea; 9 Department of Urology, Renji Hospital, School of Medicine, Shanghai Jiaotong University, Shanghai 200030, Chinazhangruiyun4337@163.com (R.-Y.Z.); 10 Department of Urology, Sun Yat-Sen Memorial Hospital, Sun Yat-Sen University, Guangzhou 510120, China; 11 Department of Urology, Sengkang General Hospital, Singapore 544886, Singapore; 12 Li Ka Shing Institute of Health Sciences, The Chinese University of Hong Kong, Hong Kong, China; 13 Department of Urology, Medical University of Vienna, 1090 Vienna, Austria

**Keywords:** robot-assisted radical cystectomy, muscle-invasive bladder cancer, bladder cancer, hydronephrosis, pre-operative assessment

## Abstract

**Simple Summary:**

This research investigates how a condition called hydronephrosis—where the kidney swells due to blocked urine flow as a result of advanced bladder cancer—affects the outcomes of a surgical procedure called robot-assisted radical cystectomy, used to remove the bladder in this group of patients. The study analysed data from multiple Asian expert centers to see if patients with hydronephrosis before surgery had different outcomes compared to those without it. Results showed that patients with hydronephrosis tended to have worse survival rates and more complications following surgery. Understanding these impacts helps doctors better predict and improve treatment outcomes for bladder cancer patients. This study’s findings can lead to more personalised and effective treatment plans, enhancing patient care.

**Abstract:**

Introduction: Robot-assisted radical cystectomy (RARC) has gained momentum in the management of muscle invasive bladder cancer (MIBC). Predictors of RARC outcomes are not thoroughly studied. We aim to investigate the implications of preoperative hydronephrosis on oncological outcomes. Patients and Methods: This study analysed data from the Asian RARC consortium, a multicentre registry involving nine Asian centres. Cases were divided into two groups according to the presence or absence of pre-operative hydronephrosis. Background characteristics, operative details, perioperative outcomes, and oncological results were reviewed. Outcomes were (1) survival outcomes, including 10-year disease-free survival (DFS) and overall survival (OS), and (2) perioperative and pathological results. Multivariate regression analyses were performed on survival outcomes. Results: From 2007 to 2020, 536 non-metastatic MIBC patients receiving RARC were analysed. 429 had no hydronephrosis (80.0%), and 107 (20.0%) had hydronephrosis. Hydronephrosis was found to be predictive of inferior DFS (HR = 1.701, *p* = 0.003, 95% CI = 1.196–2.418) and OS (HR = 1.834, *p* = 0.008, 95% CI = 1.173–2.866). Subgroup analysis demonstrated differences in the T2-or-above subgroup (HR = 1.65; *p* = 0.004 in DFS and HR = 1.888; *p* = 0.008 in OS) and the T3-or-above subgroup (HR = 1.757; *p* = 0.017 in DFS and HR = 1.807; *p* = 0.034 in OS). Conclusions: The presence of preoperative hydronephrosis among MIBC patients carries additional prognostic implications on top of tumour staging. Its importance in case selection needs to be highlighted.

## 1. Introduction

Muscle-invasive bladder cancer (MIBC) constitutes a significant proportion of bladder cancer cases worldwide, with evolving surgical management over the decades [1]. The introduction of robot-assisted radical cystectomy (RARC) has transformed the surgical treatment of MIBC by providing a minimally invasive alternative to the conventional open approach [2]. Although the oncological effectiveness of RARC has been proven by multiple studies [3,4,5], there is still a pressing need to identify prognostic factors that can aid in patient selection in the real-world clinical scenario to enhance treatment outcomes.

Hydronephrosis, indicative of ureteric obstruction, frequently occurs in advanced bladder cancer [6]. In the era of open radical cystectomy, preoperative hydronephrosis has been extensively evaluated as a prognostic marker, with findings indicating its association with advanced tumour stages and inferior survival outcomes [7]. Nonetheless, the prognostic significance of hydronephrosis in patients undergoing RARC has yet to be fully explored.

This study aims to fill this knowledge gap by utilising data from the Asian RARC Consortium, a multicentre registry that includes nine high-volume centres across Asia. Representing one of the largest real-world datasets, our research aims to investigate the effects of preoperative hydronephrosis on perioperative, pathological, and survival outcomes in patients with MIBC treated using RARC. Through identifying the prognostic value of hydronephrosis in MIBC, we aim to enhance risk stratification and patient counselling in order to refine the management of MIBC in the era of robotic surgery.

## 2. Methods and Materials

The Asian Robotic Assisted Radical Cystectomy Consortium was established in 2017, consisting of nine expert centres across Asia. It was established to explore the potential real-world benefits and morbidities of robotic-assisted radical cystectomy. This analysis encompassed consecutive cases of non-metastatic bladder cancer patients treated with RARC between 2007 and 2020. Cases lacking pre-operative information regarding the presence or absence of hydronephrosis, or with insufficient follow-up data were excluded. A standardised electronic form was utilised to collect data on patient demographics, disease specifics, operative parameters, early post-operative outcomes, recurrence, and survival data. The disease characteristics included local and systemic staging, with T (tumour) and N (nodal) stages determined through specimen review (pathological staging). Patient characteristics included age, comorbidities, and ASA status. Operative details recorded comprised the type of urinary diversion (intracorporeal or extracorporeal), console time, and estimated blood loss. Post-operative data included the length of hospital stay, instances of complications, and readmission within 30 days post-operation. Early post-operative complications, defined as events occurring within 30 days, were graded according to the Clavien-Dindo classification system. The documentation, grading, and reporting of complications in our cohort adhered to the EAU quality criteria for accurate and comprehensive reporting of surgical outcomes [8].

Hydronephrosis prior to the operation was determined based on the most recent cross-sectional imaging, either by computer tomography (CT) or magnetic resonance imaging (MRI). The cohort was divided into two groups: (1) those with no pre-operative hydronephrosis and (2) those with hydronephrosis (unilateral or bilateral). Outcomes of interest included short-term and long-term results. Perioperative outcomes included pathological outcomes and perioperative results, while long-term outcomes analysed were disease-free survival (DFS) and overall survival (OS). Disease-free survival was defined as the interval from robotic cystectomy to the first recurrence, progression of disease, metastasis, or death, whichever occurred first. Oncological outcomes and survival data were collected during each follow-up, with death recorded as all-cause mortality. Patients alive without evidence of disease were censored as of the date of the last follow-up.

For statistical analysis, established recommendations for data analysis were applied [9]. Categorical variables were presented using descriptive statistics and percentages, while continuous variables were reported as medians with interquartile ranges, or means with a standard deviation and a standard error of means. The Chi-square test and Fisher’s exact test were employed to examine categorical variables, whereas the Student’s T-test was used for continuous variables. A two-tailed *p*-value of 0.05 was considered statistically significant. A multivariate regression analysis was undertaken to identify confounding factors influencing disease-free and overall survival. Two models of multivariate regression analysis were used to evaluate the impact of hydronephrosis on (1) disease-DFS and (2) OS. These outcomes were further analysed in subgroup analyses classified by T staging (groups with T2+ and T3+ separately) to scrutinise the effect of hydronephrosis in conjunction with local tumour staging. Covariates included achieving clear surgical margins, pathological T stage, pathological N stage, tumour histology (as per WHO 2004 criteria) [10], and the use of adjuvant chemotherapy. The number of covariates satisfied the criteria for preventing model overfitting in our multivariate regression analyses [11]. Statistical analyses were performed using SPSS version 24.0 (IBM).

## 3. Results

From 2007 to 2020, a total of 536 patients underwent RARC in nine centres across Asia, with documented status concerning the presence or absence of pre-operative hydronephrosis. 20 cases had been excluded from analysis due to a lack of baseline data. The median follow-up duration was 26.0 months. Of these patients, 429 (80.0%) exhibited no pre-operative hydronephrosis, while 107 (20.0%) had hydronephrosis prior to cystectomy, with 76 (71.0%) having unilateral and 31 (29.0%) bilateral hydronephrosis.

Patient characteristics were comparable between the two groups. Age, proportions of diabetes, ASA status, and smoking status were similar. Fewer patients in the non-hydronephrotic group suffered from preoperative renal impairment (7.5% vs. 26.2% in the hydronephrotic group; defined as an estimated glomerular filtration rate of <60 mL/1.73 m^2^/kg). A slightly higher percentage of patients in the non-hydronephrotic group received neoadjuvant chemotherapy, although the difference was not statistically significant (30.8% vs. 22.4%, *p* = 0.085). Comparable proportions of patients underwent intracorporeal urinary reconstruction (48.9% vs. 57.0%, *p* = 0.68) and lymph node dissection (98.1% vs. 100%) following RARC. For pathological staging, a higher proportion of patients in the hydronephrosis group were at stage T3 and above (31.2% vs. 48.6%, *p* = 0.004), with a similar proportion being node positive (17.3% vs. 24.3%, *p* = 0.114). These details are presented in Table 1.

Table 2 outlines the perioperative outcomes related to the effects of preoperative hydronephrosis. Within the non-hydronephrotic group, pathological upstaging was noted in 125 cases (29.1%), compared to 44 cases (41.1%) in the hydronephrotic group (HR = 1.70, *p* = 0.018). Hydronephrosis was also a predictor of 30-day readmission (20.5% in the non-hydronephrotic group vs. 40.2% in the hydronephrotic group, HR = 2.692, *p* < 0.001) and severe post-operative complications with a Clavien-Dindo grade ≥ 3 (13.3% vs. 24.3%, HR = 2.095, *p* = 0.006). However, nodal upstaging, prolonged hospital stays, excessive blood loss, prolonged console times, and all-grade complications did not correlate with preoperative hydronephrosis.

**Figure 1:**
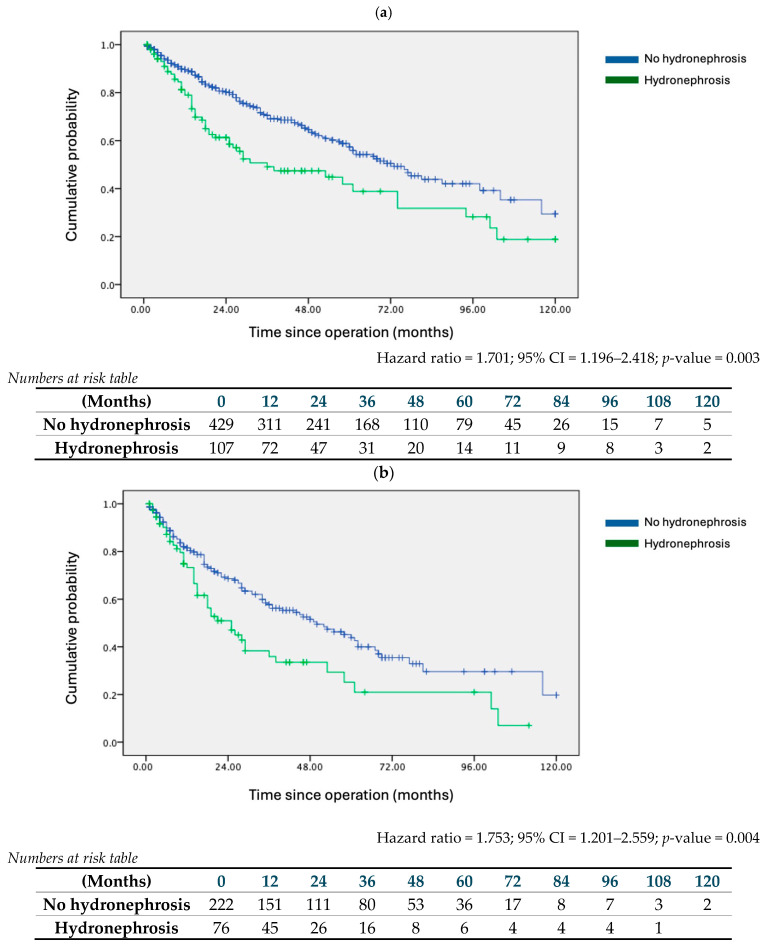

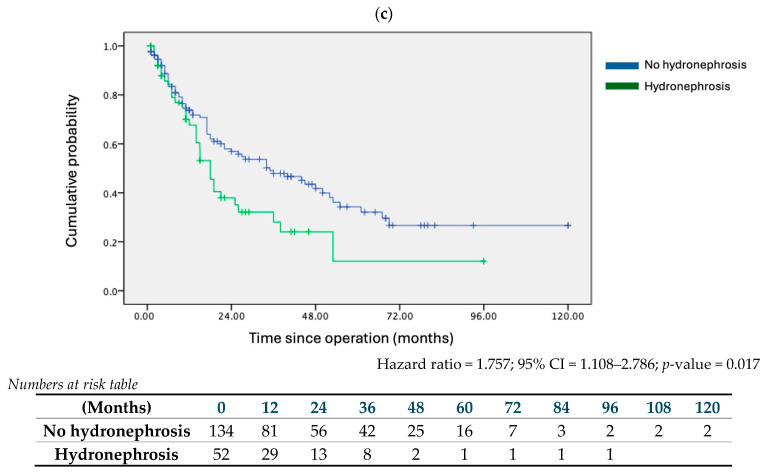
Kaplan–Meier survival curves on 10-year disease-free survival (DFS) of no hydronephrosis and hydronephrosis cohorts. (**a**) Entire cohort, (**b**) pT2+ stage or above subgroup, (**c**) pT3+ stage or above subgroup.

**Figure 2:**
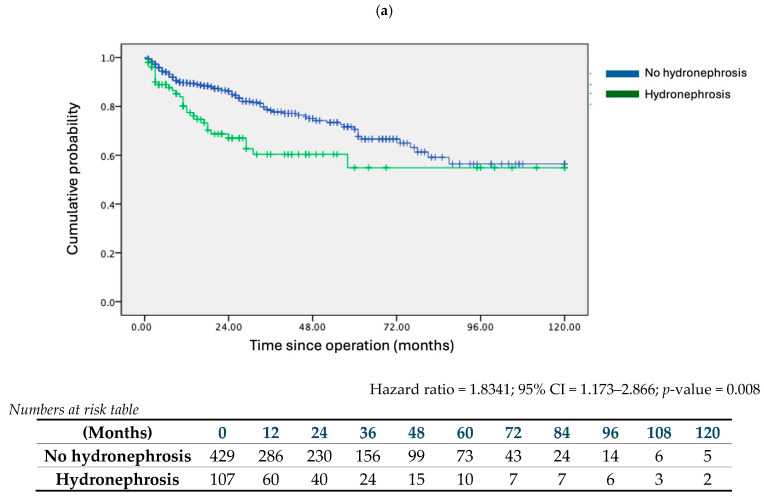

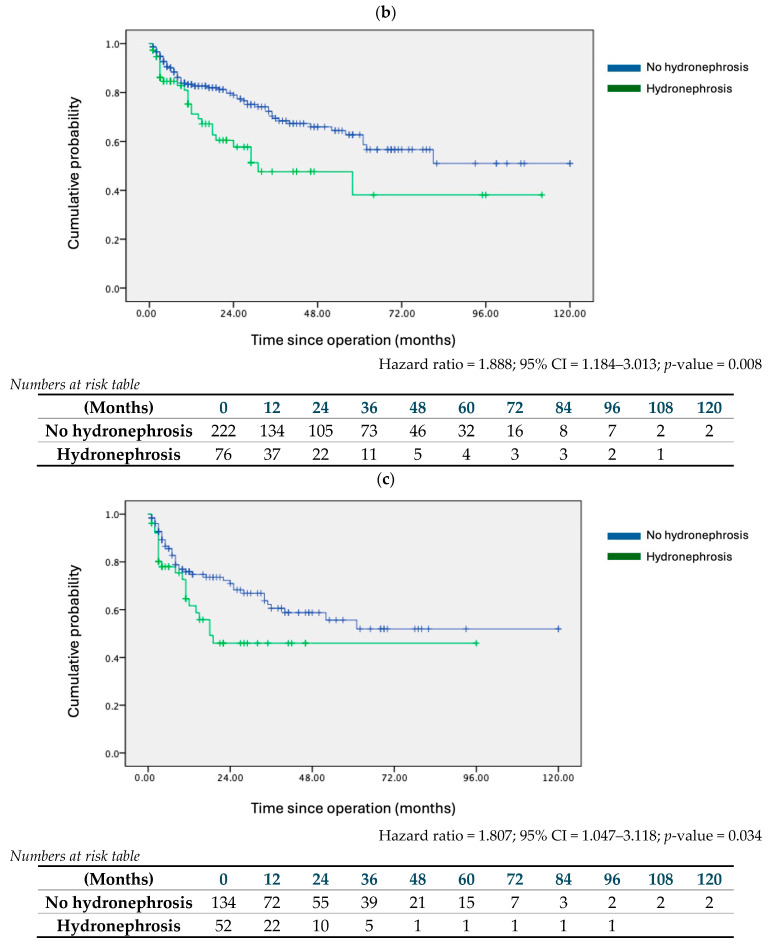
Kaplan–Meier survival curves on 10-year overall survival (OS) of no hydronephrosis and hydronephrosis cohorts. (**a**) Entire cohort, (**b**) pT2+ stage or above subgroup, (**c**) pT3+ stage or above subgroup.

The impact of preoperative hydronephrosis on DFS and OS was analysed. Kaplan-Meier survival analysis indicated that hydronephrosis was associated with worse 10-year DFS (Figure 1a) (HR = 1.701; 95% CI = 1.196–2.418; *p* = 0.003), and OS (Figure 2a) (HR = 1.8341; 95% CI = 1.173–2.866; *p* = 0.008). This association persisted in multivariate regression analysis. Alongside hydronephrosis, adjuvant therapy and high-grade tumours were significant factors adversely affecting both DFS and OS (Table 3a).

Subgroup analysis was conducted by segregating patients according to tumour T stage. In the pT2+ subgroup, hydronephrosis conferred disadvantages in both DFS (HR = 1.753; 95% CI = 1.201–2.559; *p* = 0.004) and OS (HR = 1.888; 95% CI = 1.184–3.013; *p* = 0.008) (Figure 1b and Figure 2b). Similarly, in the pT3+ subgroup, the presence of hydronephrosis correlated with worse survival outcomes (DFS: HR = 1.757; 95% CI = 1.108–2.786; *p* = 0.017; OS: HR = 1.807; 95% CI = 1.047–3.118; *p* = 0.034) (Figure 1c and Figure 2c). Multivariate analysis for both subgroups confirmed hydronephrosis as a consistent predictor of worse DFS and OS. Table 3b details the multivariate analysis for the pT2+ subgroup, noting that, apart from hydronephrosis, only an advanced pT stage was a predictor of worse outcomes. Table 3c presents the multivariate analysis for the pT3+ subgroup, indicating that both hydronephrosis and advanced pT stage were predictors of worse DFS, while hydronephrosis alone was a predictor of worse OS.

## 4. Discussion

To the best of our knowledge, this study represents the first cohort focusing exclusively on robotic-assisted radical cystectomy to report on the impact of hydronephrosis on both perioperative and long-term outcomes. Our findings indicate that pre-operative hydronephrosis correlates with adverse pathological outcomes, notably pathological upstaging. Moreover, hydronephrosis was associated with significant complications and increased 30-day readmissions. Notably, hydronephrosis emerged as a predictor of poorer survival outcomes, demonstrating worse DFS and OS compared to the non-hydronephrotic group. This association remained statistically significant after adjusting for confounders such as pT stage and adjuvant therapy and persisted across advanced disease subgroups (pT ≥ 2 and pT ≥ 3).

The current literature on the influence of hydronephrosis on the perioperative outcomes of radical cystectomy (RC) is inconclusive. Two meta-analyses conducted in 2019 attempted to consolidate recent evidence. Oh et al. analysed 24 studies involving 10,461 RC patients, revealing an association of hydronephrosis with advanced T stage (OR = 2.56), advanced N stage (OR = 2.44), and inferior OS (OR = 1.67) [7]. Additionally, Zhu et al. reviewed 13 studies and reported that hydronephrosis was linked to poorer OS (HR = 1.36) and cancer-specific survival (CSS) (HR = 1.64), but not to DFS [12]. Contrary to these findings, our study identified not only an inferior OS but also a clear DFS disadvantage in the hydronephrotic group.

The consistent observation across various studies that hydronephrosis is associated with advanced tumour T stage can be attributed to the aggressiveness of the tumour, which may compress or invade the ureteric orifice and submucosal sections of the ureters, leading to hydronephrosis [13,14,15,16]. More significantly, our study concluded that hydronephrosis provides additional prognostic information, enhancing the prediction of poorer outcomes independently of tumour T stage. This aligns with findings from a recent retrospective cohort by Du et al. [17], which demonstrated that hydronephrosis served as a marker of poorer OS in the T3-4N0M0 subgroup. Within our T3-4 subgroup, those with hydronephrosis exhibited worse DFS.

The mechanisms by which hydronephrosis leads to inferior oncological outcomes remain speculative. We propose that hydronephrosis acts as a marker of chronic obstruction from muscle-invasive bladder cancer (MIBC), leading to increased hydrostatic pressure within the collecting system [18]. This may potentially facilitate the release of circulating tumour cells (CTC) into the bloodstream. Evidence from three meta-analyses supports that CTC positivity in bladder cancer patients is predictive of inferior OS, DFS, and progression-free survival (PFS), substantiating the detrimental impact of hydronephrosis observed in our study [19,20,21].

Another hypothesis concerning hydronephrosis and its association with inferior oncological outcomes involves metastasis to the lymph nodes. Tumour chronicity, which was associated with hydronephrosis [22], could increase the likelihood of nodal micrometastases. This correlation was well-demonstrated in a series indicating that delayed cystectomy in high-risk NMIBC was associated with increased chances of lymph node metastasis [23]. Several studies had shown that preoperative hydronephrosis was related to pelvic lymph node invasion in RC specimens [6,24,25], although our current study did not find such a correlation. This may be due to the challenges of detecting micrometastases in lymph node specimens. Cuck et al. reviewed 832 lymph node samples from 61 RC patients classified as pN0 and discovered micrometastases in 3.27% of the patients using the AE1AE3 cytokeratin marker [26]. This finding suggested this supposedly pN0 cohort might actually be understaged and, consequently, undertreated, thus elucidating the inferior survival outcomes. However, diagnosing micrometastases remains a clinical challenge, as even CT or MRI achieve less than 60% sensitivity and specificity in diagnosing pelvic lymphadenopathies [27]. The potential prognosticative and predictive role of preoperative hydronephrosis—which could be readily identified in staging imaging—could not be understated.

Yet another potential explanation for the inferior oncological outcomes associated with hydronephrosis is the increased likelihood of tumour spillage during various stages of RC. It is impractical to empirically demonstrate that hydronephrotic ureters during surgery translate to challenging specimen handling and potential tumour spillage, as quantifying such spillage for research and documentation is exceedingly difficult. However, we can infer the consequences of tumour spillage. Wei et al. reported a small series of 17 RARC patients who had pelvic washes collected intraoperatively; they noted that positive cytology from these samples correlated with a higher chance of subsequent recurrence [28]. Therefore, the presence of hydronephrosis on preoperative imaging should alert surgeons to the technical challenges involved with RARC and encourage meticulous techniques to minimise pelvic micrometastases.

The limitations of the current series have to be addressed. The most apparent limitation is its retrospective nature. Despite efforts in subgroup and multivariate analysis, there could still be unidentified confounders. Selection bias cannot be fully avoided. The limitation of follow-up needs to be highlighted as well. To be specific, because of the different rates of adoption of robotic techniques in the Asian centres involved, a majority of RARC cases were performed at a later time point—when it became more widely accepted—after the initiation of the data collection of this consortium. This posed challenges to the interpretation of longer term follow-up results. Moreover, the majority of existing studies on RARC were based on the Western Caucasian population. While this dataset was acquired from multiple Asian centres, the impact of ethnic differences and hence treatment outcomes—compared with their Western counterparts—could not be adequately assessed. Another limitation is the insufficient number of cases with bilateral hydronephrosis, which precluded further analysis due to limited statistical power. On the other hand, the merits of this study should be highlighted. This series is one of the most current, utilising data from robotic cystectomies. As the trend towards increased use of robot-assisted systems in RC continues [29], our analysis offers a more contemporary perspective compared to older studies focused on open RC outcomes. In the absence of prospective series, the results demonstrated in our subgroup and multivariate analyses remain among the most robust evidence available.

## 5. Conclusions

The presence of preoperative hydronephrosis serves as a significant negative prognostic marker for bladder cancer patients undergoing robot-assisted radical cystectomy. It is imperative that surgeons consider this critical information during preoperative planning to optimise oncological outcomes and enhance patient management strategies.

## Figures and Tables

**Table 1:** Patient, disease, and operative characteristics.

	No Hydronephrosis		Hydronephrosis		*p* Value
	N	%/SD	N	%/SD	
Number of patients, %	429	80.0%	107	20.0%	
Mean age, SD	66.2	9.7	66.9	11.1	0.52
Gender (male to female)	369:60		89:18		0.46
Smoking history, %	220	51.3%	50	46.7%	0.34
Diabetes mellitus, %	96	22.4%	24	22.4%	0.94
Preoperative renal impairment, %	32	7.5%	28	26.2%	<0.001
History of abdominal surgery, %	10	2.3%	3	2.8%	0.549
History of pelvic radiotherapy, %	12	4.2%	4	2.1%	0.78
ASA, %					0.86
1	96	22.4%	26	24.3%	
2	278	64.8%	65	60.7%	
3+	55	12.8%	16	15.0%	
Neoadjuvant chemotherapy, %	132	30.8%	24	22.4%	0.085
Intracorporeal urinary reconstruction, %	234	48.9%	61	57.0%	0.68
Lymph node dissection performed, %	421	98.1%	107	100%	0.16
High grade histology, %	310	72.3%	72	67.3%	0.58
pT stage, %					0.004
T0	22	5.1%	3	2.8%	
Ta/is	106	42.7%	17	15.9%	
1	79	18.4%	11	10.3%	
2	88	20.5%	24	22.4%	
3	101	23.5%	39	36.4%	
4	33	7.7%	13	12.2%	
pN+, %	76	17.7%	26	24.3%	0.114

SD = standard deviation; ASA = American Society of Anaesthesiologist classification; pT stage = pathology tumour staging; pN+ = pathological nodal positive.

**Table 2:** Effect of hydronephrosis on early postoperative and pathological outcomes.

	HR	95% CI		*p* Value
Pathological upstaging	1.699	1.096	2.631	0.018
Nodal upstaging	1.500	0.857	2.627	0.16
Hospital stays above median	0.940	0.614	1.44	0.78
Blood loss above median	1.371	0.896	2.096	0.15
Console time above median	1.033	0.676	1.579	0.88
30-days readmission	2.692	1.702	4.256	<0.001
All grade complications	1.401	0.896	2.191	0.14
Severe complications (Clavien Dindo ≥ 3)	2.095	1.242	3.532	0.006

HR = Hazard ratio; 95% CI = 95% confidence interval.

**Table 3:** Multivariate Cox regression analysis on factors associated with disease-free and overall survival.

**(a) Entire Cohort**								
	**Disease-Free Survival**				**Overall Survival**			
	**Effect Size**	**95% CI**		***p* Value**	**Effect Size**	**95% CI**		***p* Value**
Hydronephrosis	1.701	1.196	2.418	0.003	1.834	1.173	2.866	0.008
Clear surgical margin	1.216	0.85	1.739	0.284	0.698	0.425	1.149	0.158
Adjuvant therapy	2.145	1.492	3.085	<0.001	1.954	1.221	3.126	0.005
pN+ status	1.49	1.01	2.198	0.044	1.243	0.752	2.056	0.396
High grade tumour	1.757	1.061	2.912	0.029	2.419	1.197	4.885	0.014
pT stage	1.191	1.047	1.355	0.008	1.151	0.976	1.357	0.095
**(b) pT2+ Stage or above Subgroup**								
	**Disease-Free Survival**				**Overall Survival**			
	**Effect Size**	**95% CI**		***p* Value**	**Effect Size**	**95% CI**		***p* Value**
Hydronephrosis	1.753	1.201	2.559	0.004	1.888	1.184	3.013	0.008
Clear surgical margin	1.286	0.876	1.889	0.2	0.713	0.416	1.22	0.216
Adjuvant therapy	1.411	0.946	2.105	0.091	1.402	0.85	2.313	0.185
pN+ status	1.222	0.813	1.835	0.335	0.999	0.601	1.66	0.996
High grade tumour	0.554	0.278	1.104	0.093	0.626	0.27	1.454	0.276
pT stage	pT2 as reference				pT2 as reference			
pT3	1.586	1.014	2.483	0.044	2.054	1.18	3.577	0.011
pT4	2.905	1.665	5.066	<0.001	2.882	1.386	5.991	0.005
**(c) pT3+ Stage or above Subgroup**								
	**Disease-Free Survival**				**Overall Survival**			
	**Effect Size**	**95% CI**		***p* value**	**Effect Size**	**95% CI**		***p* Value**
Hydronephrosis	1.757	1.108	2.786	0.017	1.807	1.047	3.118	0.034
Clear surgical margin	1.014	0.651	1.579	0.951	0.581	0.312	1.083	0.087
Adjuvant therapy	1.141	0.724	1.798	0.57	1.33	0.763	2.32	0.315
pN+ status	1.122	0.72	1.749	0.61	0.849	0.488	1.476	0.561
High grade tumour	0.68	0.269	1.72	0.415	0.69	0.246	1.935	0.481
pT stage	pT3 as reference				pT3 as reference			
pT4	1.784	1.132	2.812	0.013	1.305	0.71	2.402	0.392

95% CI = 95% confidence interval; pT stage = pathology tumour staging; pN+ = pathological nodal positive.

## Data Availability

Data is available upon request via submitting a request to the corresponding author.
